# The CDH patient perspective journey

**DOI:** 10.3389/fped.2023.1052422

**Published:** 2023-02-21

**Authors:** Beverley Power

**Affiliations:** Management Committee, CDH UK – The Congenital Diaphragmatic Hernia Charity, King’s lynn, United Kingdom

**Keywords:** CDH, Congenital Diaphragmatic Hernia, patient journey, outcomes, pediatrics, healthcare, neonatal, transition of care

## Abstract

**Background:**

Congenital Diaphragmatic Hernia is a malformation of the diaphragm resulting in ongoing clinical symptoms and problems. Mortality remains high, particularly where there are other issues involved. Tracking a patient throughout their lifetime to understand the full impact on health and function is challenging. CDH UK is a registered charity supporting anyone affected by CDH. It has over 25 years of experience and a broad range of patient experience and knowledge.

**Aims:**

To develop a patient journey with timepoints of significance.

**Methods:**

We studied our own data and looked at what we already knew from publications and medical advisors. We recruited a focus group, plotted out stages and timepoints through their “lived” experiences using the Team Idea Mapping method. We then compared these experiences to our own data, to identify the common issues in daily life and care.

**Outcome:**

We have developed a patient journey through the eyes of the patient and turned it into a patient friendly infographic. This can be used as a tool to help understand the CDH Journey throughout a patient's lifetime. CDH UK has already used this to create a first prototype of a mobile application. It has also further helped to recognize areas of patient concern and to improve services and resources.

**Discussion:**

This can be used as a basis for care and research, including standards, benchmarking, transition and helping improvements in healthcare, education, family life and social settings. Potentially holding clues as to the etiology and pathology of the condition and an opportunity to further explore theories and unanswered questions. It may help improve counselling and bereavement care, resulting in better general and mental health outcomes.

## Introduction

Congenital Diaphragmatic Hernia is a malformation of the diaphragm resulting in long lasting clinical symptoms and problems, that is still poorly understood in terms of long-term outcome (1, 2).

One of the main difficulties of the care of patients with this condition is tracking their progress throughout their lifetime to understand the pathology, to preserve, prevent, and improve health, with the aim of effecting a good quality of life. Transition of care from the pediatric care setting to an adult care setting can be problematic, with little or no planning in the pediatric setting. This often results in poor health outcomes later in life due in part to a lack of knowledge and experience of the condition, particularly in the General Practice healthcare setting.

CDH UK is a registered UK charity that supports anyone affected by CDH, or who has an interest in Congenital Diaphragmatic Hernia. It was founded in 1994 as an informal support group and registered as a charity with the Charity Commission for England and Wales in 2004 (registration number 1106065) and in Scotland in 2011 (registration number SC042410). The services and resources of CDH UK are accessed by thousands of individuals meaning that the charity has access to a large cohort of patients and carers, which results in a good overview and understanding of patient experiences, needs and priorities. This is mainly acquired due to voluntary patient reported outcomes.

In 2014 CDH UK began thinking about developing a mobile application for patients and families to facilitate patient reported outcomes and to enable them to input and retrieve day to day data. This culminated in approaching developers specializing in mobile applications for patient use. In 2016 we were asked to provide a patient journey by a chosen developer to plot out the relevant time points for the basis of the mobile application, but we realized that there was no published patient journey for CDH and certainly not from the perspective of the patient.

It became clear that not only did we need to know the journey for our mobile application development, but for other reasons such as improving our support services and resources and for research and study too: particularly with the advancement of data collection and technology. Research will most likely benefit from understanding the whole lifespan of a patient journey. Healthcare professionals often do not have information on their patient for a lifetime, as they either discharge in early life, or lose track of the patient during transition, or for other reasons. Neither do they have “lived” experience of Congenital Diaphragmatic Hernia. Therefore, information on a patient's journey will be beneficial for planning transition of care and understanding any potential future health problems that their patient may experience.

This poses the question “What does the CDH patient perspective Journey look like?”

We aimed to develop a patient perspective Journey, mapping out timepoints of significance in a patient's lifetime referencing points of care, wellbeing and social aspects that depicts what happens; what we know happens, what we think we know happens and what we would like to see happen, and at what timepoints in life.

## Methods

The CDH Patient Perspective journey was created using a mixed method of Qualitative and Quantitative research carried out in 4 stages.

### Stage 1

We studied our own historical data that was less than 10 years old and that was collected through various means as follows:

1. Online surveys using Survey Monkey™.
2. Posts and comments on our Social Media accounts and groups
3. Face to face conversations at Get Together meetings
4. Online events
5. Support line calls
6. Emails into the support inbox

The data analyzed was derived from various ages of individuals falling in to three categories:

1. Parents (biological or non-biological)
2. Carers (Any person involved in the care of the patient or their family other than parents)
3. Patients (the person suffering with a Congenital Diaphragmatic Hernia or eventration)

The aim was to look for problems reported, or common requests for support, to enable us to understand common themes in care, health, or quality of life issues.

We also considered what we already knew from publications, attending conferences and from discussions with our medical advisors and Patrons.

**Figure 1:**
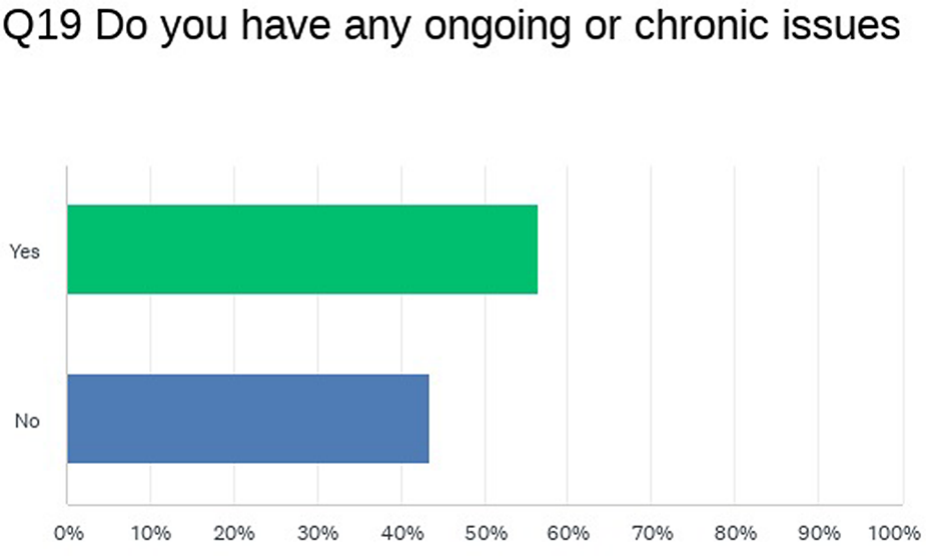
Analyzed data from CDH UK transition from child to adult services survey question 19.

The example below is of data captured and analyzed from twenty-three adult respondents of one question within a survey regarding transition of care (Figure 1).

### Stage 2

**Figure 2:**
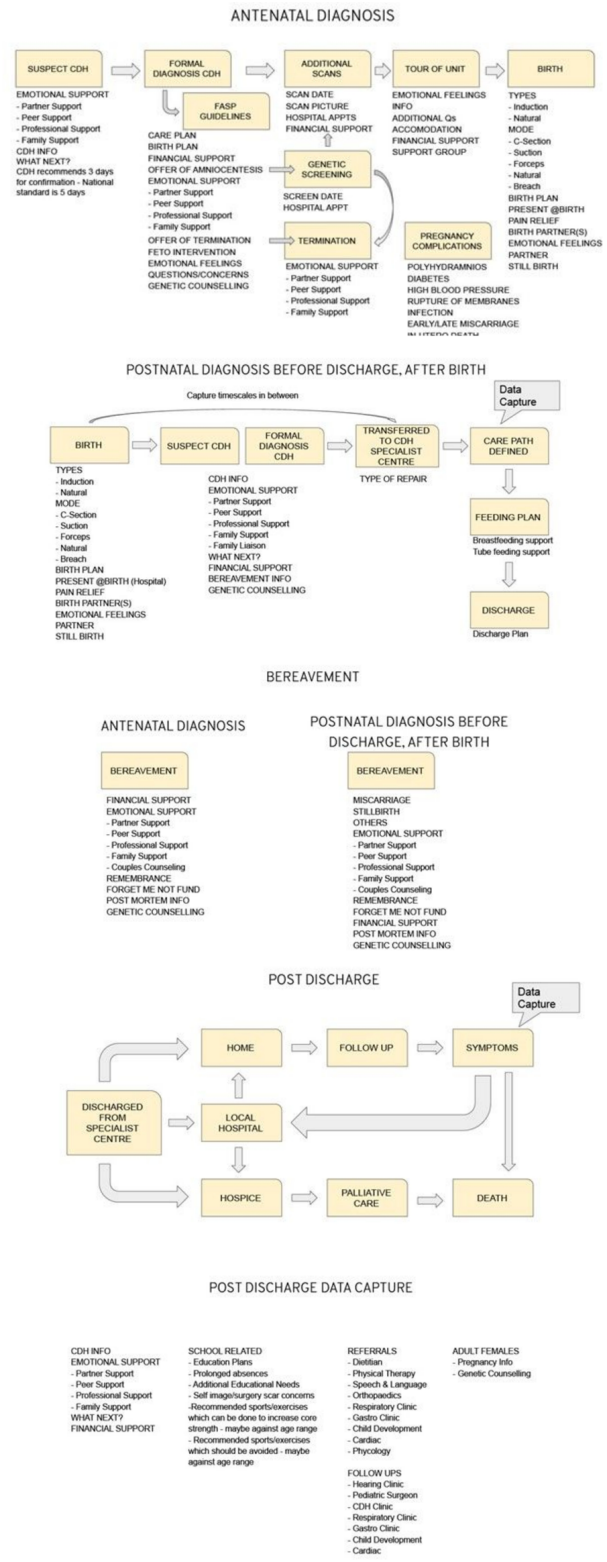
Flow Map.

A focus group of thirteen parents and other family members of mixed sex and ages was recruited by approaching our members. Nine members of the group met face to face (two virtually) for a full afternoon workshop to plot out the various stages and timepoints through their “lived” experiences using the Team Idea Mapping Method (3), which allowed us to create a flow map of different scenarios (Figure 2). This included Antenatal diagnosis, postnatal diagnosis before discharge, after birth, bereavement, and post discharge. We also added a list of data capture points at the end of the flow map.

### Stage 3

We compared the flow map to experiences of the patients and families that we have supported over the years using data derived from Stage 1. We considered examples of patient reported outcomes of care that were below what the patients or parents expected and examples of good practices and good care according to patients and parents, but not necessarily according to literature, or local healthcare guidelines.

**Figure 3:**
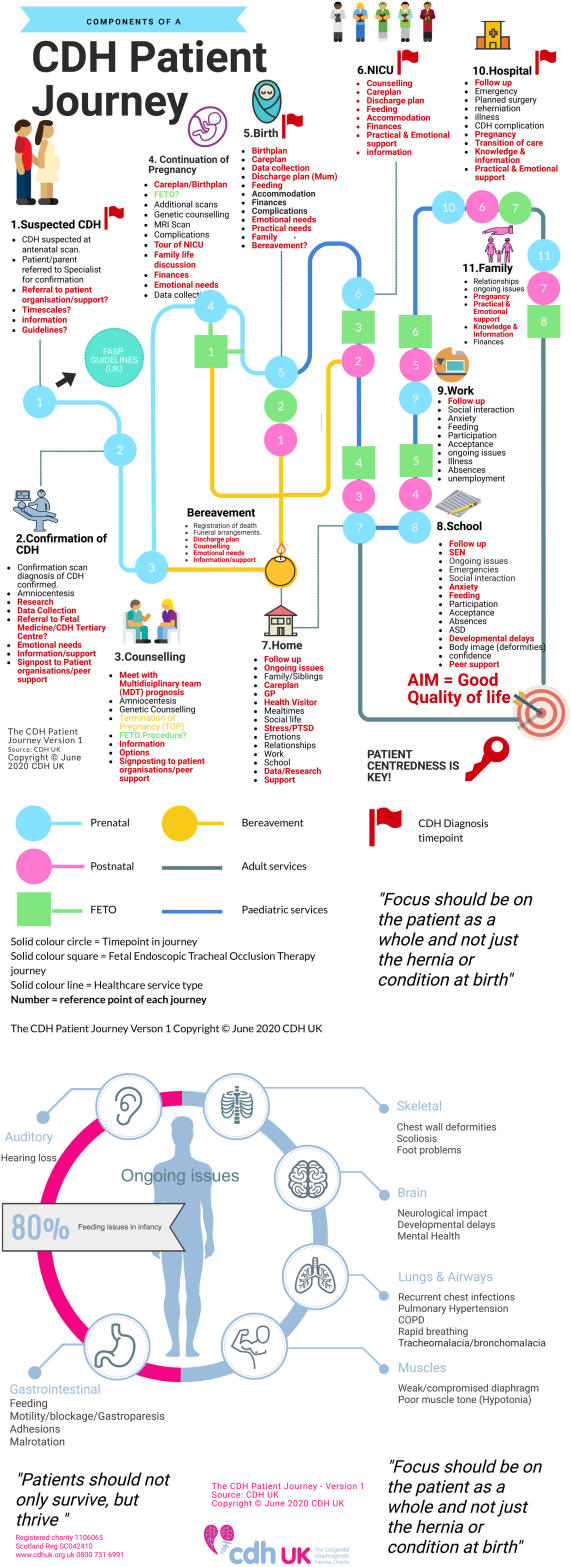
Components of a CDH patient journey.

We created a visual using a paid creative license titled “Components of a CDH Patient Journey” (Figure 3). This visual was presented at our Great Get Together online event in June 2020, but feedback proved that this visual is too complex to follow and could not be used as an infographic for lay persons.

**Figure 4:**
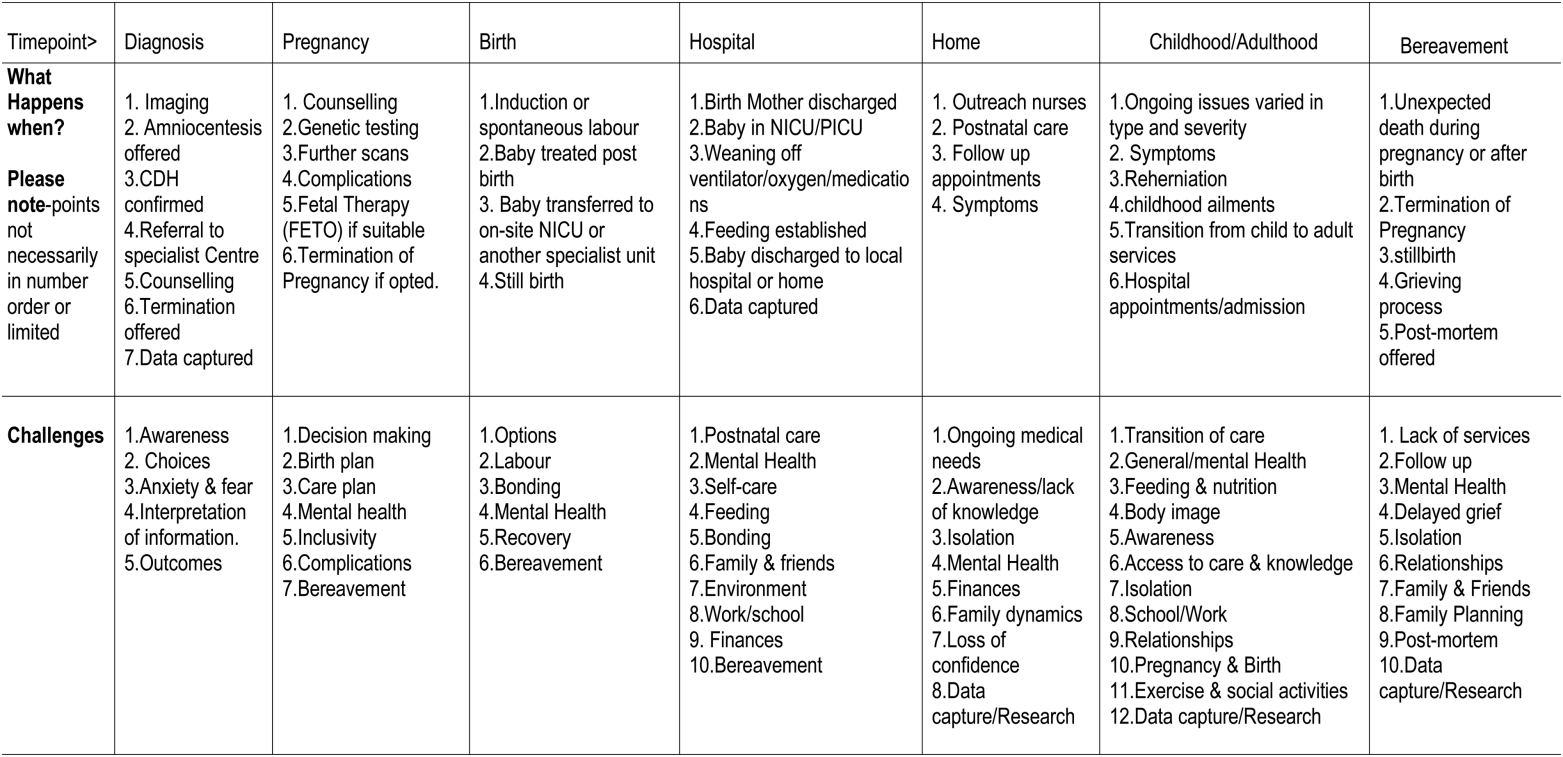
Table of amendments.

We asked a group of over twenty families, patients and caregivers to carry out a further review of the first visual (Figure 3) to help to reduce the text and simplify the visual. The group provided a table of amendments (Figure 4).

### Stage 4

**Figure 5:**
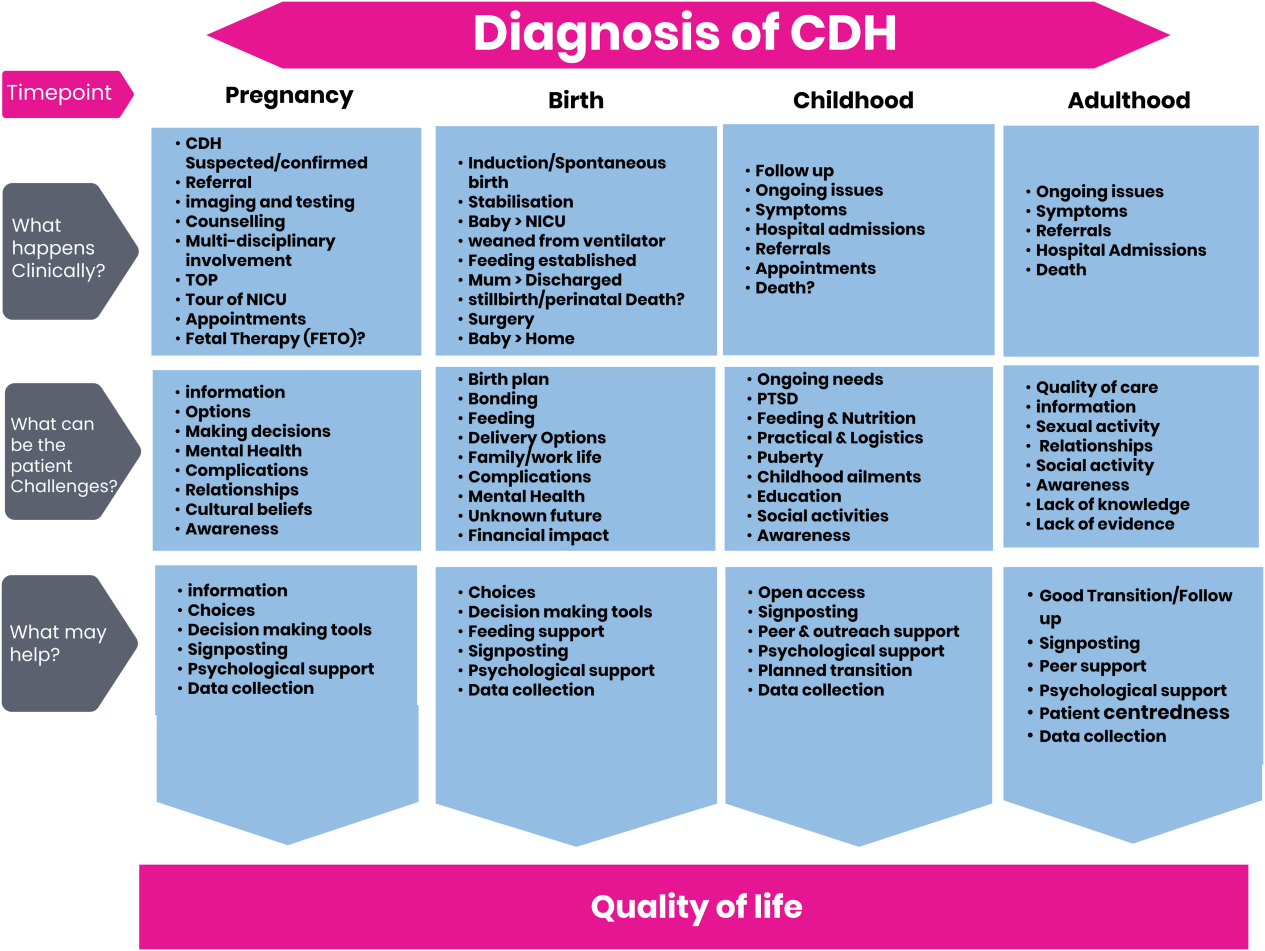
CDH Patient Journey chart.

**Figure 6:**
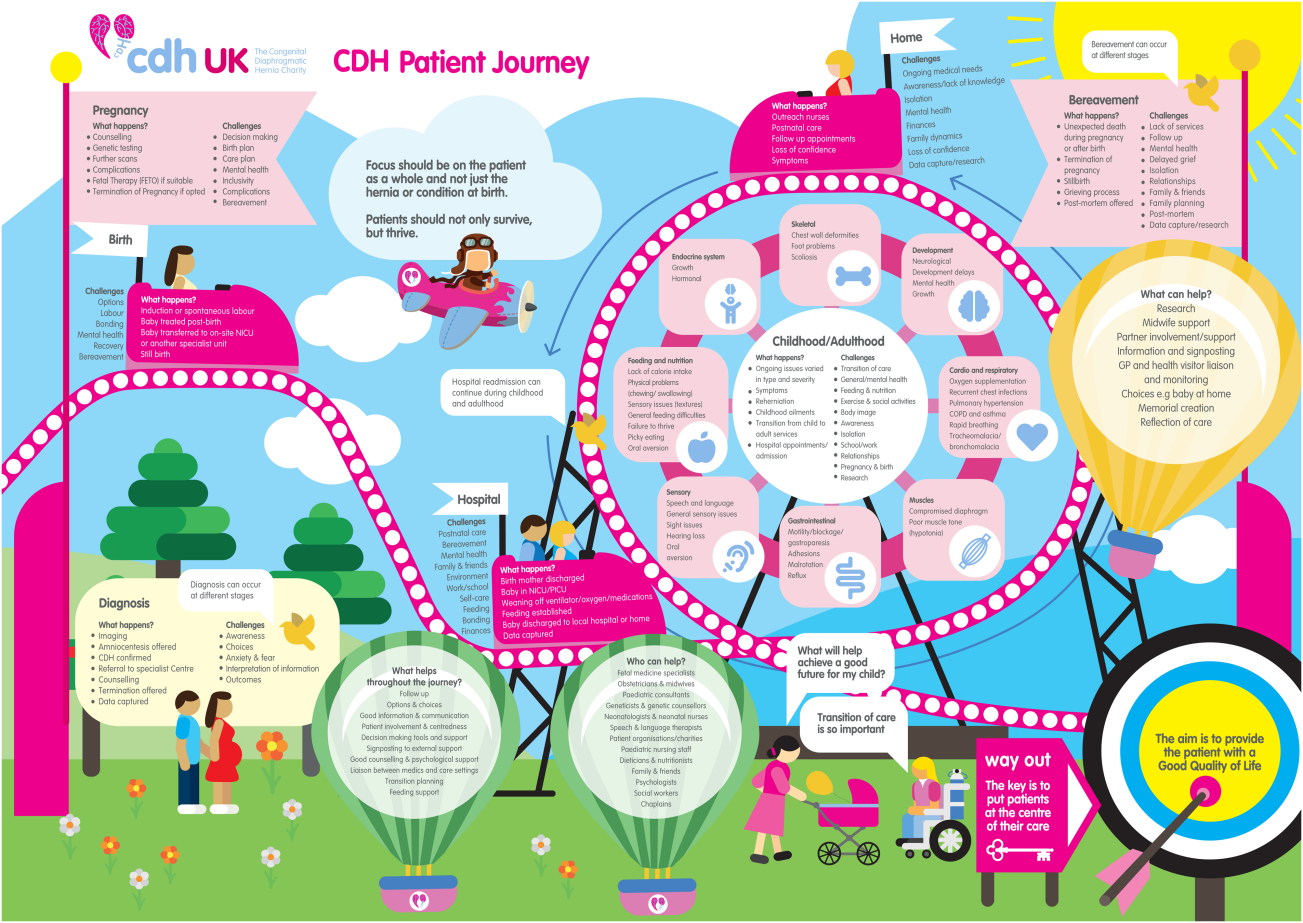
CDH Patient Journey V1 infographic.

An infographic chart (Figure 5) and patient friendly infographic (Figure 6) was developed using the feedback from this further group review. The consensus was reached by the described four stages that involved patients, family members, experts in CDH and graphic designers. The patient friendly infographic is depicted as “A rollercoaster journey” as this is often how parents and patients describe it. This is simpler in its form than the earlier version depicted in The Components of a CDH Patient Journey (Figure 3).

## Ethical considerations

We considered all ethical issues during our research. No ethical approvals were sought as no personally identifiable data is used in our data or reporting. All focus groups were created voluntarily and all participants in our surveys and focus groups were free to withdraw at any time without having to give a reason. There were no payments or recompense given for face-to-face focus group meetings as these took place alongside other meetings and so require no additional out of pocket expense to the group members.

## Results

We have identified what the CDH patient Journey looks like for the parents and patient in terms of their lived experience with an antenatal or postnatal diagnosis of CDH, or a diagnosis in later life. Using the information derived from the research stages, we have developed a visual infographic of the patient perspective journey that can be used by patients, caregivers, and researchers (for example), due to the various formats that can be produced from the flow chart. This will provide an insight and help the reader to better understand the CDH Patient Journey throughout a patient's lifetime and to make them aware of the potential health, social, economic, and logistical issues that may impact the patient or family. We recognize that the journey has some limitations; there are a spectrum of case presentations seen with Congenital Diaphragmatic Hernia, with not all cases presented or diagnosed during pregnancy, at birth, or soon after, and so ongoing issues may not be diagnosed early enough to impact on outcomes. Also, with new management strategies and treatments this journey may change, and so periodic review is necessary to reflect up to date patient experiences. In addition, the data is derived from personal experiences which may have been influenced by various other factors.

## Discussion

The developed patient journey chart can be used as a basis for care in the UK and beyond, including developing standards, benchmarking, and improvements in care. We also hope that it can be useful for research and will be an instigator for new translational research (4).

The Patient Journey is an ever evolving one, due in part to advances in treatments, care, support, and technology. We therefore realize that the CDH Patient Perspective journey must be reviewed regularly and suggest every two years to ensure it is as accurate and as relevant as possible. We have developed a strategy to ensure that the information that CDH UK produces is of a certain standard and quality and that its lifecycle is maintained.

This CDH Patient Perspective journey may also hold clues as to the etiology and pathology of the condition and could harness an opportunity to further explore theories and unanswered questions. There is also evidence of improved survival rates in severe Congenital Diaphragmatic Hernia (5) and left sided cases (6), which will impact on health services. It may also serve as a basis for the improvement in counselling and bereavement care, resulting in better mental health outcomes for both patients and families.

We have used this patient journey already in its raw form, to create a prototype for a mobile application. We hope in the short term the current Patient Journey Version 1 will serve as a useful support resource for patients, families, and caregivers and in the long term will help with research.

## Data Availability

The original contributions presented in the study are included in the article/Supplementary Material, further inquiries can be directed to the corresponding author.

## References

[1] Zimmer J Puri P. Congenital diaphragmatic hernia. In: Puri P, editors. Pediatric surgery. Berlin, Heidelberg: Springer (2017). p. 797–815. 10.1007/978-3-642-38482-0_57-1

[2] Tan JK Banton G Minutillo C Hall GL Wilson A Murray C Long-term medical and psychosocial outcomes in congenital diaphragmatic hernia survivors. Arch Dis Child. (2019) 104(8):761–7. 10.1136/archdischild-2018-316091 30877092

[3] Goswami B Mahajan R Jain A Koner BC. Team idea mapping method: A brainstorming session for enhancing problem-solving skills in postgraduate medical biochemistry students as assessed by self-efficacy. J Med Sci & Res. (2021) 3(2):74–8. 10.25259/AUJMSR_23_2021

[4] Friedmacher F Pakarinen MP Rintala RJ. Congenital diaphragmatic hernia: a scientometric analysis of the global research activity and collaborative networks. Pediatr Surg Int. (2018) 34(9):907–17. 10.1007/s00383-018-4304-7 30019129

[5] Gien J Kinsella JP Behrendt NJ Zaretsky MV Galan HL Liechty KW. Improved survival for infants with severe congenital diaphragmatic hernia. J Perinatol. (2022) 42:1189–94. 10.1038/s41372-022-01397-3 35461332

[6] Yang MJ Fenton S Russell K Yost CC Yoder BA. Left-sided congenital diaphragmatic hernia: can we improve survival while decreasing ECMO? J Perinatol. (2020) 40(6):935–42. 10.1038/s41372-020-0615-3 32066841

